# TLR2 signal influences the iNOS/NO responses and worm development in C57BL/6J mice infected with *Clonorchis sinensis*

**DOI:** 10.1186/s13071-017-2318-y

**Published:** 2017-08-07

**Authors:** Qing-Li Yang, Ji-Qing Shen, Zhi-Hua Jiang, Yun-Liang Shi, Xiao-Ling Wan, Yi-Chao Yang

**Affiliations:** 1 Guangxi Key Laboratory for Viral Hepatitis Prevention and Control, Guangxi Zhuang Autonomous Region Center for Disease Prevention and Control, Nanning, 530028 Guangxi People’s Republic of China; 2 Department of Parasitology, Guangxi Medical University, Nanning, 530021 Guangxi People’s Republic of China

**Keywords:** *Clonorchis sinensis*, Inducible nitric oxide synthase, Toll-like receptor 2, C57BL/6 J mice

## Abstract

**Background:**

Although the responses of inducible nitric oxide synthase (iNOS) and associated cytokine after *Clonorchis sinensis* infection have been studied recently, their mechanisms remain incompletely understood. In this study, we investigated the effects of toll-like receptor 2 (TLR2) signals on iNOS/nitric oxide (NO) responses after *C. sinensis* infection. We also evaluated the correlations between iNOS responses and worm development, which are possibly regulated by TLR2 signal.

**Methods:**

TLR2 wild-type and mutant C57BL/6 J mice were infected with 60 *C. sinensis* metacercariae, and the samples were collected at 30, 60, 90 and 120 days post-infection (dpi). The total serum NO levels were detected using Griess reagent after nitrate was reduced to nitrite. Hepatic tissue samples from the infected mice were sliced and stained with hematoxylin and eosin (HE) to observe worm development in the intrahepatic bile ducts. The iNOS mRNA transcripts in the splenocytes were examined by real time reverse transcriptase polymerase chain reaction (qRT-PCR), and iNOS expression was detected by immunohistochemistry.

**Results:**

Developing *C. sinensis* juvenile worms were more abundant in the intrahepatic bile ducts of TLR2 mutant mice than those of TLR2 wild-type mice. However, no eggs were found in the faeces of both mice samples. The serum levels of total NO significantly increased in TLR2 mutant mice infected with *C. sinensis* at 30 (*t*_(5)_ = 2.595, *P* = 0.049), 60 (*t*_(5)_ = 7.838, *P* = 0.001) and 90 dpi (*t*_(5)_ = 3.032, *P* = 0.029). Meanwhile, no changes occurred in TLR2 wild-type mice compared with uninfected controls during the experiment. The iNOS expression in splenocytes showed unexpected higher background levels in TLR2 mutant mice than those in TLR2 wild-type mice. Furthermore, the iNOS mRNA transcripts in splenocytes were significantly increased in the TLR2 wild-type mice infected with *C. sinensis* at 30 (*t*_(5)_ = 5.139, *P* = 0.004), 60 (*t*_(5)_ = 6.138, *P* = 0.002) and 90 dpi (*t*_(5)_ = 6.332, *P* = 0.001). However, the rising of iNOS transcripts dropped under the uninfected control level in the TLR2 mutant mice at 120 dpi (*t*_(5)_ = -9.082, *P* < 0.0001). Both total NO and iNOS transcripts were significantly higher in the TLR2 mutant mice than those in the TLR2 wild-type mice at 30 (*t*_(5)_ = 3.091/2.933, *P* = 0.027/0.033) and 60 dpi (*t*_(5)_ = 2.667/6.331, *P* = 0.044/0.001), respectively. In addition, the remarkable increase of iNOS expressions was immunohistochemically detected in the splenic serial sections of TLR2 wild-type mice at 30 and 60 dpi. However, the expressions of iNOS were remarkably decreased in the splenocytes of both TLR2 wild-type and mutant mice at 120 dpi.

**Conclusions:**

These results demonstrate that TLR2 signal plays an important role in the regulation of iNOS expression after *C. sinensis* infection. TLR2 signal is also beneficial to limiting worm growth and development and contributing to the susceptibility to *C. sinensis* in which the iNOS/NO reactions possibly participate.

**Electronic supplementary material:**

The online version of this article (doi:10.1186/s13071-017-2318-y) contains supplementary material, which is available to authorized users.

## Background

*Clonorchis sinensis*, commonly known as the Chinese liver fluke, is a food-borne parasite and the causative agent of clonorchiasis in humans [1, 2]. Clonorchiasis is mainly prevalent in Asian countries, including South Korea, China, northern Vietnam and far-eastern Russia [3, 4]. The adult worms of *C. sinensis* can survive for a long period in human bile ducts and cause severe lesions in the hepatic biliary, and promote progression of cholangiocarcinoma (CCA) and hepatic carcinoma [2, 5]. Nitric oxide (NO) formation and nitrosation may contribute to the development of liver fluke-associated carcinogenesis [6]. NO is a product of L-arginine conversion to L-citrulline by nitric oxide synthase (NOS). Among the three major isoforms of NOS, inducible NOS (iNOS) is most associated with the pathophysiology of inflammatory diseases [7]. In our recent studies, we confirmed iNOS induction and associated cytokine expression in BALB/c mice after *C. sinensis* infection [8, 9].

The expression of the iNOS gene is particularly regulated by activated nuclear factor-κB (NF-κB) [10, 11]. Furthermore, a previous study demonstrated that excretory-secretory products (ESPs) from *C. sinensis* induce the expression of proinflammatory cytokines, IL-1β, IL-6 and iNOS/NO responses in human CCA cells in a NF-κB-dependent manner [12]. The results of our recent work also revealed that *C. sinensis* ESPs promote NF-κB activation and NO production in RAW264.7 macrophages [13]. However, the mechanisms of the iNOS/NO responses after *C. sinensis* infection are not completely understood.

Toll-like receptors (TLRs) are transmembrane proteins that recognize pathogen-associated molecular patterns (PAMPs) of multiple pathogens, including bacteria, viruses, fungi and parasites [14, 15]. The TLR family is crucial to microbial elimination and homeostasis and plays an important immunoregulatory role [16, 17]. In particular, TLR2 and TLR4 are upregulated in the hepatocytes of BALB/c mice and downregulated in C57BL/6 mice during *Trypanosoma cruzi* infection. The results of this study further demonstrated that TLR2, TLR4 and TLR9 all play important roles in modulating injured livers from these two types of mice during *T. cruzi* infection [18]. Additionally, a recent study reported that *C. sinensis* infection promotes the upregulation of TLR2 and TLR4 transcripts, which are strongly expressed in the cytoplasms and endothelial cell membranes, fibroblasts and biliary epithelium cells in C3H/He mice. This finding suggests that TLR2 and TLR4 might be involved in immune responses during *C. sinensis* infection [19].

Several studies reported that many strains of mice, including FVB/NJ (FVB) [20–22], BALB/c [8, 9, 20–23], and C3H/He [24] are susceptible to *C. sinensis*. By contrast, C57BL/6 mice are resistant to *C. sinensis* infection [22, 23]. Among the TLRs responsible to immune regulation, TLR2 plays the most important role in host defense [25, 26]. Further study indicated the importance of TLR2 signal for hepatic immune and nonimmune cells in attenuating strong inflammatory liver response during *T. cruzi* acute infection [27]. In this experiment, we investigated iNOS/NO expression and development of *C. sinensis*, which are possibly influenced by TLR2 signaling during the infection, in natural ‘low responders’ C57BL/6 J mice.

## Methods

### Animals and experimental infection

Specific pathogen-free TLR2 wild-type C57BL/6 J (B6) and TLR2 mutant B6 (TLR2^mut/mut^) mice (all female) at 5 weeks of age were obtained from the Nanjing Biomedical Research Institute of Nanjing University (NBRI, China) and further bred in the barrier facility at the Guangxi Laboratory Animal Centre (GLAC, China). The *C. sinensis* metacercariae were collected from *Pseudorasbora parva* through a previously described method [28]. Groups of six animals were inoculated with 60 *C. sinensis* metacercariae in 400 μl of 0.9% NaCl by oral gavage. Mice gavaged with 0.9% NaCl were used as uninfected controls. All mice were fed a sterile food and water until sample collection 30, 60, 90 and 120 days post-infection (dpi).

### Adult worm growth visualization and egg detection

The liver tissues were collected and fixed with 4% paraformaldehyde and then embedded in paraffin for histological detection. The embedded tissues were cut into 4 μm-thick sections in continuous succession and then placed on adhesion microscope slides (CITOGLAS, Haimen, China). The sections were baked at 60 °C for 30 min and then soaked with dimethylbenzene (3 times for 5 min), absolute ethanol (2 times for 5 min), 95% ethanol (2 times for 5 min), 70% ethanol (1 times for 5 min) and pure water (2 times for 5 min) successively for dewaxing and dehydration. The sections were stained with eosin (Biotech Well, Shanghai, China) for 5 min at room temperature and then washed with clean water. The cleaned slides were counterstained with hematoxylin solution (Biotech Well) at room temperature for 10 min and washed with pure water to stop reaction. Finally, the slides were soaked in blue-appearing solution (Biotech Well) to elicit a blue color. The development of adult worms in the intrahepatic bile ducts was observed under a microscope.

Stool samples were collected from each infection group every other day and were mixed together every 20 days. Eggs were counted by the Kato-Katz egg counting technique [29]. For perfect blending, 1 ml of pure water was added into 1 g of stool of each sample. The stool samples were then thoroughly mixed after they were completely humidified [9]. Using standard 41.7 mg templates, we prepared triplicate Kato-Katz thick smears from each stool sample. Eggs of *C. sinensis* were then observed under a light microscope.

### Total NO detection

The serum levels of total NO were detected with total nitric oxide assay kit (Nitrate/Nitrite Assay Kit; Beyotime, Shanghai, China) according to the manufacturer’s protocol. Briefly, nitrate was reduced to nitrite by NADPH-dependent nitrate reductase and quantified with Griess reagent. The level of total NO was calculated through the detection of the total quantity of nitrate and nitrite. Finally, the reactions were stopped, and the OD values were recorded at 540 nm with an endpoint reading (Multiskan GO, ThermoFisher Scientific, Waltham, MA, USA). Nitrite concentrations were calculated with reference to a calibration curve prepared with a standard solution (2–80 μM) of sodium nitrite.

### Immunohistochemistry (IHC)

The spleens were taken and fixed with 4% paraformaldehyde for the immunohistochemical analyses of iNOS expression. The liver tissue sections were deparaffinized in xylene and rehydrated through graded alcohol as described above. The sections were then incubated with endogenous peroxidase blocker (Biotech Well) at room temperature for 15 min. After washing in pure water three times for 5 min, the sections were treated with antigen retrieval reagents (Biotech Well) at 95–100 °C for 20 min. After washing in PBS (pH 7.4), the sections were incubated with goat serum immunol staining blocker (Biotech Well) at room temperature for 2 h. After removing the liquid, the sections were incubated with 1:250 diluted mouse mAb [NOS-IN] to iNOS (Abcam) at 4 °C overnight. After washing, the sections were sequentially incubated with biotin-conjugated goat antimouse IgG (Biotech Well) and streptavidin-biotin-peroxidase complex (SABC; Biotech Well) at room temperature for 20 min. The slides were washed again and then stained with AEC horseradish peroxidase color development kit (Biotech Well) at room temperature for 10 min. The slides were washed with water, air-dried and counterstained with hematoxylin solution (Biotech Well) according to the procedure described above.

### Total RNA and DNA extraction

Splenocytes were washed with PBS (pH 7.4), resuspended in 200 μl of sample protector for RNA/DNA (TaKaRa, Dalian, China), and stored at -80 °C until RNA extraction. Total RNA and DNA samples were extracted from the splenocytes with TRIzol Reagent (Invitrogen, Carlsbad, CA, USA) through a single step. The RNA was isolated from the aqueous phase of the homogenized samples according to the manufacturer’s protocol, and the RNA pellets were dissolved in 15 μl of RNase-free water. Genomic DNA was collected from the remaining interphase and organic phase from the initial homogenate after RNA extraction and precipitated with ethanol according to the manufacturer’s protocol. The air-dried DNA pellets were dissolved in 400 μl of 8 mM NaOH.

### Polymerase chain reaction (PCR) and real-time quantitative reverse transcriptase PCR (qRT-PCR)

Wild-type and mutant TLR2 genes were detected by PCR according to the protocol of NBRI. Briefly, PCR amplification was carried out in the presence of 1× *Ex Taq* buffer, 2 mM of Mg^2+^, 0.2 mM of each dNTP, 0.4 μM of each primer (Additional file 1: Table S1) and 0.025 U/μl of *Ex Taq* in a 25 μl reaction (TaKaRa, Dalian, China). PCR was performed with the following parameters: 1 cycle of 94 °C for 5 min and 35 cycles of a denaturing step of 94 °C for 30 s, an annealing step of 58 °C for 30 s, an extension step of 72 °C for 45 s, and a final extension of 72 °C for 3 min. Amplicons were inspected on 1.5% agarose regular (TaKaRa) with premixed GelRed™ nucleic acid gel stain (Biotium, Inc., Hayward, CA, USA).

The expression levels of iNOS mRNA were quantified by qRT-PCR. First, cDNA was synthesized by reverse transcription of total RNA with PrimeScript™ II 1st Strand cDNA Synthesis Kit (TaKaRa) according to the manufacturer’s instructions. Specific primers used in PCR and real-time assays were synthesized by Sangon Biotech Co., Ltd. (Shanghai, China) (Additional file 1: Table S1). Subsequent real-time PCR operations were performed SYBR® Fast qPCR Mix (TaKaRa). Briefly, modified *Taq* HS mutant, DNA intercalated dye SYBR Green I, Tli RNaseH, dNTP mixture, Mg^2+^ and 0.4 μM of gene specific primers, 2 μl of cDNA and 0.4 μl of 50× ROX reference dye II were combined in a final volume of 20 μl. Amplification and data acquisition were performed with an Applied Biosystems 7500 Real-Time PCR system running on V2.0.6 software (Life Technologies, Carlsbad, CA, USA) and using the following cycling parameters: predenaturation at 95 °C for 30 s, 40 cycles of amplification at 95 °C for 5 s, and annealing and extension at 60 °C for 30 s. Melt curve analysis was performed from 65 °C to 95 °C to determine the melting temperatures (Tm) (Additional file 1: Table S1) of the identified specific DNA product populations. The mRNA transcript of iNOS was quantified with RT-PCR assay based on the slope of standard curve described previously [8, 30]. Serial diluted cDNA samples were used as standards for the preparation of standard curves and acquisition of slopes. The iNOS and β-actin mRNA expression levels were quantified and expressed as Q_T_ and Q_R_, respectively. The relative level of iNOS mRNA expression was calculated with reference to β-actin and expressed as adjusted value (Q_T_/Q_R_).

### Statistical analyses

Data were presented as the mean ± standard error of the mean, SEM. The significant difference levels of total NO and relative levels of iNOS mRNA expression were compared by paired-sample *t*-tests. Calculations were performed on IBM SPSS Statistics version 19.0 software. Comparisons were considered significant at *P* ≤ 0.05 and highly significant at *P* ≤ 0.01 or *P* ≤ 0.001.

## Results

### Worm development in the intrahepatic bile ducts of C57BL/6 J mice

**Fig. 1:**
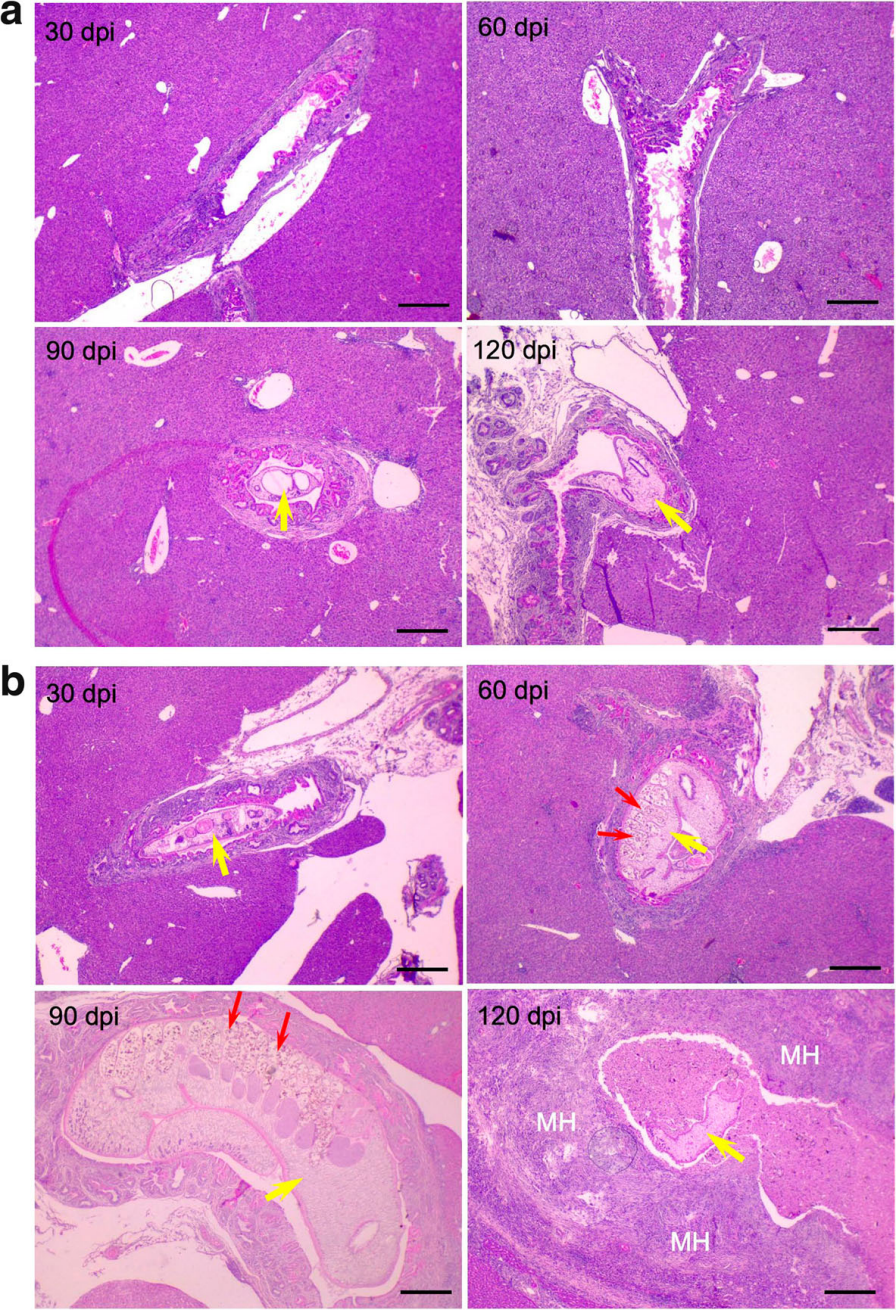
Worms developing in the intrahepatic bile duct of C57BL/6 J mice after *C. sinensis* infection. **a** TLR2 wild-type mice. **b** TLR2 mutant mice. Slides were treated by HE stain (× 35). The arrows in yellow and red highlight the developing worms and their immature uteri, respectively. *Abbreviations*: dpi, days post-infection; MH, malignant hyperplasia of the intrahepatic bile duct. The figure shows one representative biopsy out of six. *Scale*-*bars*: 1000 μm

Serial histological sections were observed under a light microscope to observe the developmental level of *C. sinensis* from juvenile to adult in the intrahepatic bile ducts. No developing worm was found in the hepatobiliary ducts of the TLR2 wild-type mice at 30 and 60 dpi, and only few immature juvenile worms were observed at 90 and 120 dpi (Fig. 1a). By contrast, different levels of development of *C. sinensis* were observed, from juvenile to nearly mature worms, in the TLR2 mutant mice during the experiment. Juvenile worms were present in the intrahepatic bile ducts at 30 dpi, grew continually at 60 dpi and developed to mature worms with well-developed uterus at 90 dpi. However, the well-developed worms became atrophied and died in the intrahepatic bile ducts with severe hyperplasia in the TLR2 mutant C57BL/6 J mice at 120 dpi (Fig. 1b).

Fecal examination was also performed to further confirm adult worm development and determine the intensity of infection on the basis of the presence of *C. sinensis* eggs. However, no eggs were found in the faeces of the TLR2 wild-type and mutant mice after infection.

### Infection of *C. sinensis* increases total NO levels in TLR2 mutant mice

**Fig. 2:**
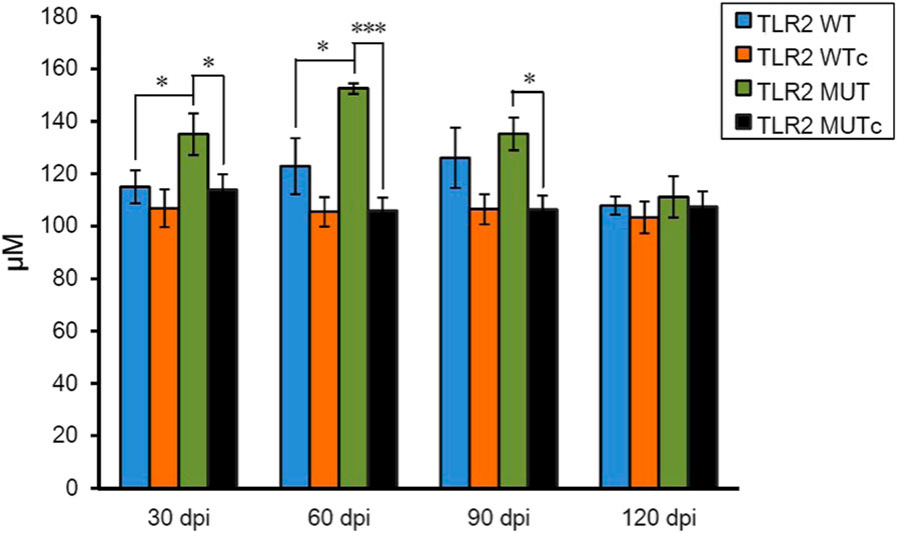
Quantification of total NO in the serum of *C. sinensis*-infected C57BL/6 J mice. The levels of total NO were quantified through the measurement of nitrate and nitrite and expressed as μM with SEM bars. Data represent the mean of six samples from each group. *Abbreviations*: WT, wild type; WTc, wild type uninfected control; MUT, mutant; MUTc, mutant uninfected control; dpi, days post-infection. *P < 0.05, ***P = 0.001

Sera were collected from the C57BL/6 J mice to measure nitrate and nitrite, the relatively stable metabolites of NO, and determine the total NO levels in C57BL/6 J mice after *C. sinensis* infection. No significant difference was observed between the background total NO levels of TLR2 wild-type and mutant mice. Furthermore, *C. sinensis* infection did not affect the total NO levels in TLR2 wild-type mice during the experiment. However, the TLR2 mutant mice showed increased levels of total NO after *C. sinensis* infection. The levels were significantly different from those in uninfected controls at 30 (*t*_(5)_ = 2.595, *P* = 0.049), 60 (*t*_(5)_ = 7.838, *P* = 0.001), and 90 dpi (*t*_(5)_ = 3.032, *P* = 0.029). Compared with TLR2 wild-type mice, TLR2 mutant mice demonstrated remarkable higher total NO levels after *C. sinensis* infection, especially at 30 (*t*_(5)_ = 3.091, *P* = 0.027) and 60 dpi (*t*_(5)_ = 2.667, *P* = 0.044). Both the TLR2 wild-type and TLR2 mutant mice demonstrated a slight decrease of total NO levels to nearly normal values at 120 dpi (Fig. 2).

### *Clonorchis sinensis* affects iNOS mRNA transcription in mouse splenocytes

**Fig. 3:**
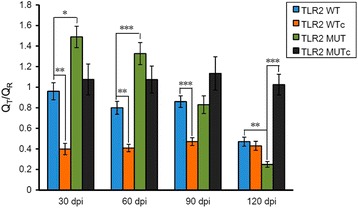
INOS mRNA transcription in the splenocytes of C57BL/6 J mice. The transcript levels of iNOS were detected using qRT-PCR and normalized with reference to β-actin gene. The relative level of iNOS transcription was expressed as adjusted value (Q_T_/Q_R_) with SEM bars. *Abbreviations*: WT, wild type; WTc, wild type uninfected control; MUT, mutant; MUTc, mutant uninfected control; dpi, days post-infection. *P < 0.05, **P < 0.01, ***P ≤ 0.001

We also examined the iNOS gene transcription in the splenocytes of C57BL/6 J mice. The iNOS transcripts were significantly increased in *C. sinensis*-infected TLR2 wild-type mice compared with those in the uninfected controls at 30 (*t*_(5)_ = 5.139, *P* = 0.004), 60 (*t*_(5)_ = 6.138, *P* = 0.002) and 90 (*t*_(5)_ = 6.332, *P* = 0.001) dpi. Furthermore, iNOS transcripts gradually decreased to the background level at 120 dpi. The transcript levels of iNOS were also increased in the TLR2 mutant mice at 30 and 60 dpi, but did not show significant differences compared with those in the uninfected controls. However, iNOS transcript levels gradually decreased at 90 dpi and dropped under the uninfected control level at 120 dpi (*t*_(5)_ = -9.082, *P* < 0.0001). Overall, the relative levels of iNOS transcripts were significantly higher in TLR2 mutant mice than in TLR2 wild-type mice at 30 (*t*_(5)_ = 2.933, *P* = 0.033) and 60 dpi (*t*_(5)_ = 6.331, *P* = 0.001). Afterwards, they were nearly in the same level at 90 dpi and underwent unexpected significant changeover at 120 dpi (*t*_(5)_ = -4.866, *P* = 0.005) (Fig. 3).

### Immunohistochemical analysis of iNOS expression in the splenocytes of mice

**Fig. 4:**
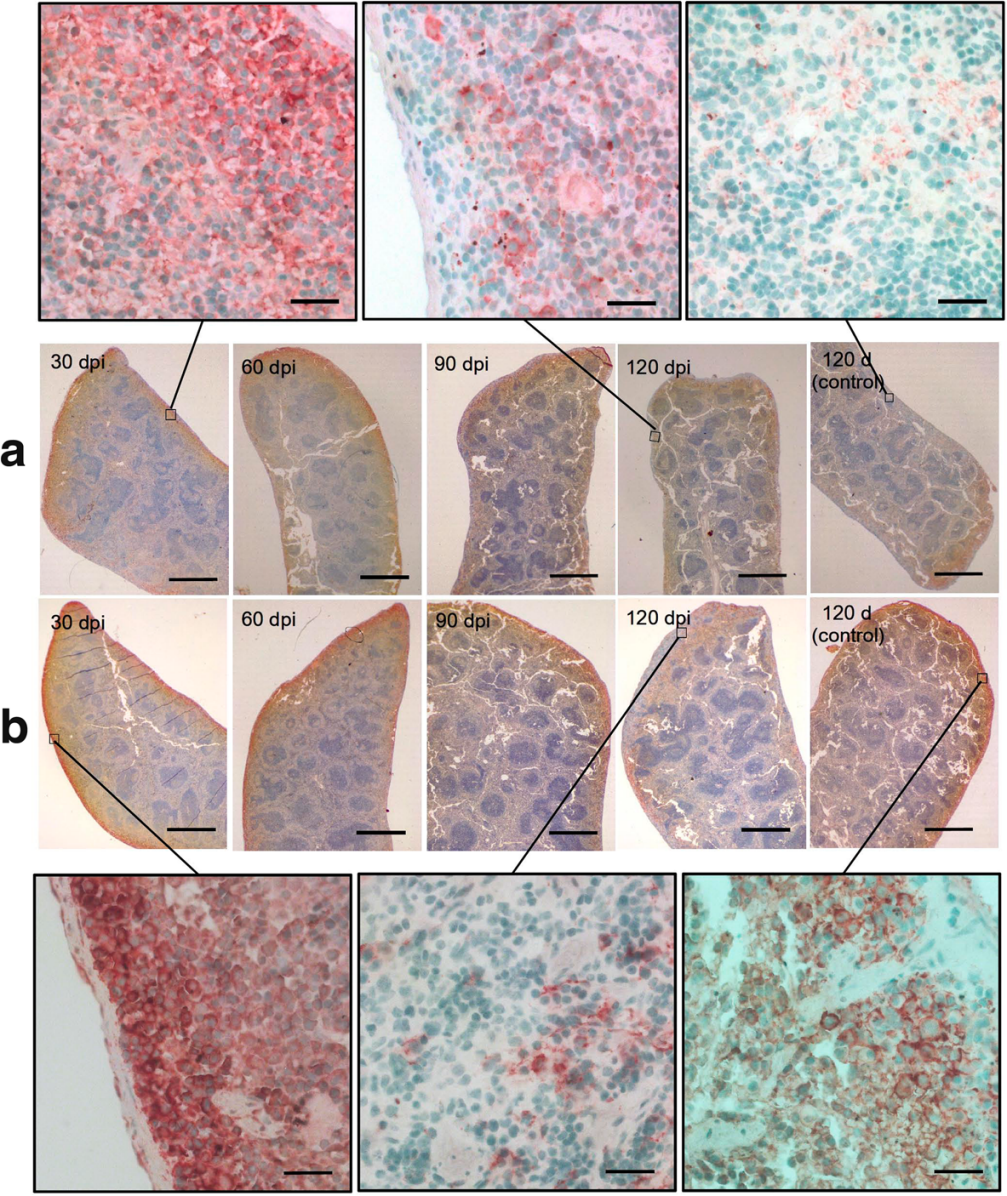
INOS expression in the splenocytes of C57BL/6 J mice after *C. sinensis* infection. The TLR2 wild-type (**a**) and TLR2 mutant (**b**) mice were inoculated with 60 *C. sinensis* metacercariae. The serial slices of the spleens were incubated with iNOS mAb, HRP-conjugated secondary antibodies, and SABC by sequence, and then processed with AEC color development reagent to show the iNOS expression in the cytoplasm with red color. Finally, the slices were counterstained with hematoxylin to display the splenocyte nuclei with blue. The figure shows one representative biopsy out of six. The uninfected normal mice were used as control. *Abbreviation*: dpi, days post-infection. *Scale-bars*: middle panels, 1300 μm; upper and lower panels, 100 μm

We also detected the iNOS expression in the spleen of C57BL/6 J mice after *C. sinensis* infection by immunohistochemical assay. Remarkable expressions of iNOS was found in the splenocytes of the TLR2 wild-type mice infected with *C. sinensis* at 30, 60, 90 and 120 dpi compared with those of the uninfected normal mice. A high background level of iNOS expression was detected accordingly in the splenocytes of the TLR2 mutant mice compared with those of the TLR2 wild-type mice. Meanwhile, stronger iNOS expressions were detected in TLR2 mutant mice at 30 and 60 dpi and gradually weakened at 90 and 120 dpi (Fig. 4).

## Discussion

Studies have demonstrated that the strains and genetic background of mice are responsible for the susceptibility to *C. sinensis* infection. C57BL/6 J mice were believed to have the lowest susceptibility to *C. sinensis* infection compared with FVB, BALB/c and C3H/He mice [20–24]. In this study, we comparatively analysed the developing processes of *C. sinensis* and the iNOS/NO responses in TLR2 wild-type and mutant C57BL/6 J mice after the infection. Both the TLR2 wild-type and mutant mice were successfully infected with *C. sinensis* and showed a remarkable increase of specific IgG antibodies (data not shown). We confirmed that C57BL/6 J mice are resistant to *C. sinensis* infection in vivo, which was consistent to the result previously reported [23]. We also found that TLR2 mutation increased the susceptibility to *C. sinensis* infection in C57BL/6 J mice. A lot of developing juvenile worms with different maturity was observed in the intrahepatic bile ducts of TLR2 mutant mice, whereas very few of them were found in the TLR2 wild-type mice during the experiment. Recently we found that *C. sinensis* were able to mature and release eggs in BALB/c mice [9]. In this study, fecal examination was also performed by modified Kato-Katz egg counting technique [9, 29] to further confirm the egg production capacities of the worms. However, no eggs were found in the faeces from both TLR2 wild-type and mutant mice after the infection. Thus, we believe that the ‘acquired’ susceptibility by TLR2 mutation is insufficient to promote *C. sinensis* maturation and subsequent egg production in C57BL/6 J mice. In contrast to *C. sinensis*-infected BALB/c mice, which presented extensive pathological lesions in the hepatobiliary tissues with periductal inflammatory cell infiltration, ductular hyperplasia, fibrosis and hepatic spotty necrosis [9], TLR2 wild-type C57BL/6 J mice presented no apparent lesions after being infected with *C. sinensis*. However, severe pathological damage presenting massive hepatic necrosis, as well as malignant intrahepatic bile ducts, were observed in the TLR2 mutant mice. The reason of this condition induced by TLR2 mutation in the ‘low responders’ C57BL/6 J mice is unclear.

In our previous studies, we found that iNOS is extensively expressed in the splenocytes, liver dendritic cells, Kupffer cells, sinusoidal endothelial cells and biliary epithelial cells of BALB/c mice after *C. sinensis* infection. Furthermore, the iNOS expression induced by *C. sinensis* could not attribute to the dominance transcripts of IFN-γ, IL-12p35 and IL-18 in splenocytes [8, 9]. Many studies demonstrated that certain cytokine responses induced by *T. cruzi* infection were associated with pathogen clearance, mortality and liver pathologies. In C57BL/6 J mice, abundant pro-inflammatory cytokines induced by *T. cruzi* infection possibly improve resistance to infection [18, 27]. Thus, iNOS/NO responses induced by *C. sinensis* might also be associated with hepatobiliary tissue damages in TLR2 mutant C57BL/6 J mice and their susceptibility to infection.

To further confirm the influence of iNOS expression on the susceptibility to *C. sinensis* infection, we used the natural ‘low susceptible’ C57BL/6 J mice and their TLR2 mutant counterparts as hosts for *C. sinensis*. According to the data supported by NBRI, both the TLR2 wild-type and TLR2 mutant mice hold a homogeneous genetic background, except for the seven single nucleotide polymorphism sites (rs33208334, rs13483055, rs29209692, rs3709624, rs3724876, rs6212539 and rs3023342). The amplified PCR products for wild-type and mutant TLR2 were 499 and 334 bp, respectively (Additional file 2: Figure S1). Notably, the baseline levels of iNOS expression in the splenocytes of both mice types were remarkably higher than that in BALB/c mice [8, 31]. The iNOS expression levels in the TLR2 wild-type and TLR2 mutant mice were remarkably induced by *C. sinensis* at 30 and 60 dpi, and the latter had higher iNOS expression level than the former in the corresponding period. High levels of iNOS/NO responses are favorable to *C. sinensis* development in the TLR2 mutant mice at the early stage of infection. The C57BL/6 J mice possibly acquired ‘high susceptibility’ to *C. sinensis* infection through iNOS/NO responses caused by TLR2 mutation.

NO is generally highly reactive and potent antipathogenic molecule that can kill intracellular pathogens, including viruses [32, 33] and some bacteria [34]. Furthermore, NO production is considered is unfavorable to the development of the adult and juvenile parasitic worms, including *Trichinella spiralis*, in ‘low responder’ C57BL/6 and ‘high responder’ BALB/c mice [35]. However, the results of this study demonstrated that high levels of iNOS/NO response induced *C. sinensis* growth and development and may thus have increased the susceptibility of TLR2 mutant C57BL/6 mice to *C. sinensis* infection.

Studies demonstrated that the anti-inflammatory cytokines, IL-10 and TGF-β, were presumably related to susceptibility to infection of mice and development of worms in different mice strains. Compared with BALB/c mice, FVB mice is a more favorable host for *C. sinensis* on the basis of cyst formation in bile ducts because they have increased production of Th2-associated anti-inflammatory cytokines, including IL-4, IL-5, IL-13, IL-10 and TGF-β [21]. Recently, Zhang et al. also demonstrated that IL-10 and TGF-β transcripts in livers of C57BL/6 mice are not induced by *C. sinensis* infection, although they are dramatically increased in BALB/c- and FVB-infected mice [22]. Another study [27] reported that hepatic leukocytes from *T. cruzi*-infected C57BL/6 mice produce higher amounts of pro-inflammatory cytokines than BALB/c mice, while IL-10 and TGF-β are only released by hepatic leukocytes from BALB/c. In addition, the stimulation of TLR2 signaling decreased pro-inflammatory cytokine production and increased TGF-β levels in purified hepatic leukocytes from C57BL/6 mice infected with *T. cruzi*. Thus, the increased levels of IL-10 and TGF-β might correlate with host susceptibility to parasitic infection. In the present study, we found that high iNOS/NO expression levels caused by TLR2 mutation contribute to *C. sinensis* infection. High iNOS/NO expression levels may also influence the balance between the pro-inflammatory cytokine and TGF-β/IL-10 levels in both TLR2 wild-type and TLR2 mutant mice after *C. sinensis* infection. However, the regulation network comprising iNOS/NO, pro-inflammatory cytokines and TGF-β/IL-10 during *C. sinensis* infection is unclear.

As primary members of pattern-recognition receptors (PRRs), TLRs can distinguish PAMPs from different pathogens and promote iNOS production by inducing the activation of various transcription factors, including NF-κB [16, 34, 36]. Several TLRs, such as TLR2, TLR3, TLR4 and TLR9, are known to correlate with iNOS/NO expression induced by various PAMPs from pathogens [32, 37–44]. TLR2 is vital to immune systems because it regulates parasitic damage in hosts [19, 26, 27]. In fact, TLR4 and TLR9 pathways, other than the TLR2 signal, can promote iNOS/NO production and play an important role in modulating injured livers from BALB/c and C57BL/6 mice during parasitic infection [18, 27]. During *Schistosoma japonicum* infection, TLR2 and TLR4 promoted opposite adaptive immune responses, including T cell activation and cytotoxic gene expression, which led to different infection outcomes [45]. In addition, the balance between the induction of immunity and tolerance to *Schistosoma mansoni* infection can be regulated by other PRRs, including C-type lectins, in concerted action with TLRs [46, 47]. Thus, integrated PRR signals, apart from TLR2 signal alone, have regulatory effects on iNOS/NO response and development of *C. sinensis* in C57BL/6 mice.

PAMP molecules from parasites are distinct from traditional microbial PAMPs both in composition and effective model [15]. Notably, lipid moieties carried by pathogens are critical for pathogen identification with TLR2 [48, 49]. *Schistosoma mansoni* lipid fractions containing lysophosphatidylserine and diacylphosphatidylserine, which stimulate TLR2 signal, are functionally different from known bacterial TLR2 ligands, such as PAM3CSK4 and MALP-2 [50, 51]. Some studies obtained insights into PAMPs in *C. sinensis* ESPs [12]. In one of our previous study, compounds with varying polarities were identified from *C. sinensis* ESPs [52]. These compounds are different with respect to NO induction and NF-κB activation in RAW264.7 mouse macrophages [13]. We also found that iNOS expression dropped rapidly in TLR2 mutant C57BL/6 mice at 120 dpi, and dead worms were generally observed in the histological sections. Thus, complicated components released from *C. sinensis* possibly interact with PRRs, including TLR2, and thus influence iNOS/NO responses and participate in their regulatory networks.

Our results indicated that TLR2 signals are critical to the induction of iNOS/NO responses in C57BL/6 mice after *C. sinensis* infection. However, contrary to our expectations, TLR2 signaling had a negative rather than a positive effect on iNOS/NO expression at the early stage of infection (before 60 dpi), although this condition was changed at the later stage (120 dpi). The phases of iNOS/NO responses might correlate with the development and growth of worms. Meanwhile, other signals induced by PAMPs from *C. sinensis* possibly promote iNOS/NO production by counteracting TLR2-mediated corresponding responses. Furthermore, TLR2 coordinating with other PRRs and interacting with PAMPs from *C. sinensis* may influence host iNOS/NO responses and susceptibility to infection.

## Conclusions

We found that the TLR2 mutant is contributes to the enhancement of iNOS/NO response at the early stage of *C. sinensis* infection and to worm growth and development in the intrahepatic bile ducts of C57BL/6 J mice. TLR2 signal was demonstrated to correlate with iNOS/NO response regulation and thus might improve defense against *C. sinensis* infection.

## Additional files

Additional file 1: Table S1. Primers used in PCR and qRT-PCR assays. (DOCX 14 kb)

Additional file 2: Figure S1. Testification of TLR2 gene in C57BL/6 J mice by PCR. **a** PCR amplified products (499 bp) for TLR2 wild type. **b** PCR amplified products (334 bp) for TLR2 mutant. The data showed half samples of all. N, PCR no DNA template controls; M, 100 bp DNA ladder. (PDF 41 kb)

## Abbreviations

dpi: Days post-infection
ESPs: Excretory-secretory products
IHC: Immunohistochemistry
iNOS: Inducible nitric oxide synthase
NF-κB: Nuclear factor-κB
NO: Nitric oxide
OD: Optical density
PAMPs: Pathogen associated molecular patterns
PRRs: Pattern-recognition receptors
qRT-PCR: Quantitative real-time reverse transcriptase polymerase chain reaction
SABC: streptavidin-biotin-peroxidase complex
TLR: Toll-like receptor
Tm: Melting temperature
