# Supplementary material for: TLR2 signal influences the iNOS/NO responses and worm development in C57BL/6J mice infected with Clonorchis sinensis
Source: Parasit Vectors. 2017 Aug 7;10:379. doi: 10.1186/s13071-017-2318-y (PMC5547496; doi:10.1186/s13071-017-2318-y)
Supplement: Supplementary file 1 — Primers used in PCR and qRT-PCR assays. (DOCX 14 kb) [file 13071_2017_2318_MOESM1_ESM.docx]

**Table S1 Primers used in PCR and qRT-PCR assays**

| specificity accession primers sequences specific product product reference  number size (bp) Tm (°C) |
| --- |
| TLR2 NC_000069.6 WT 5’- ACGAGCAAGATCAACAGGAGA-3’ gDNA 499 N/A [1]  MUT 5’-GGGCCAGCTCATTCCTCCCAC-3’ 334 N/A  Common 5’-CTTCCTGAATTTGTCCAGTACA-3’  iNOS NM_010927 F 5’-GTTCTCAGCCCAACAATACAAGA -3’ cDNA 127 84.5 [2]  R 5’-GTGGACGGGTCGATGTCAC -3’  β-actin NM_007393 F 5’-GGCCGGGACCTGACAGACTACCTC -3’ cDNA 90 84.8 [3]  R 5’-GTCACGCACGATTTCCCTCTCAGC -3’ |

**References:**

1. Reference to the gene identification protocol supplied by NBRI.

2. Tsutsuki H, Yahiro K, Suzuki K, Suto A, Ogura K, Nagasawa S, et al. Subtilase cytotoxin enhances *Escherichia coli* survival in macrophages by suppression of nitric oxide production through the inhibition of NF-κB activation. Infect Immun. 2012; 80:3939–51.

3. Gerard HC, Wang Z, Whittum-Hudson JA, El-Gabalawy H, Goldbach-Mansky R, Bardin T, et al. Cytokine and chemokine mRNA produced in synovial tissue chronically-infected with *Chlamydia trachomatis* and *C. pneumoniae*. J Rheumatol. 2002;29:1827–35.
