# Supplementary figures and images for: TLR2 signal influences the iNOS/NO responses and worm development in C57BL/6J mice infected with Clonorchis sinensis
Source: Parasit Vectors. 2017 Aug 7;10:379. doi: 10.1186/s13071-017-2318-y (PMC5547496; doi:10.1186/s13071-017-2318-y)

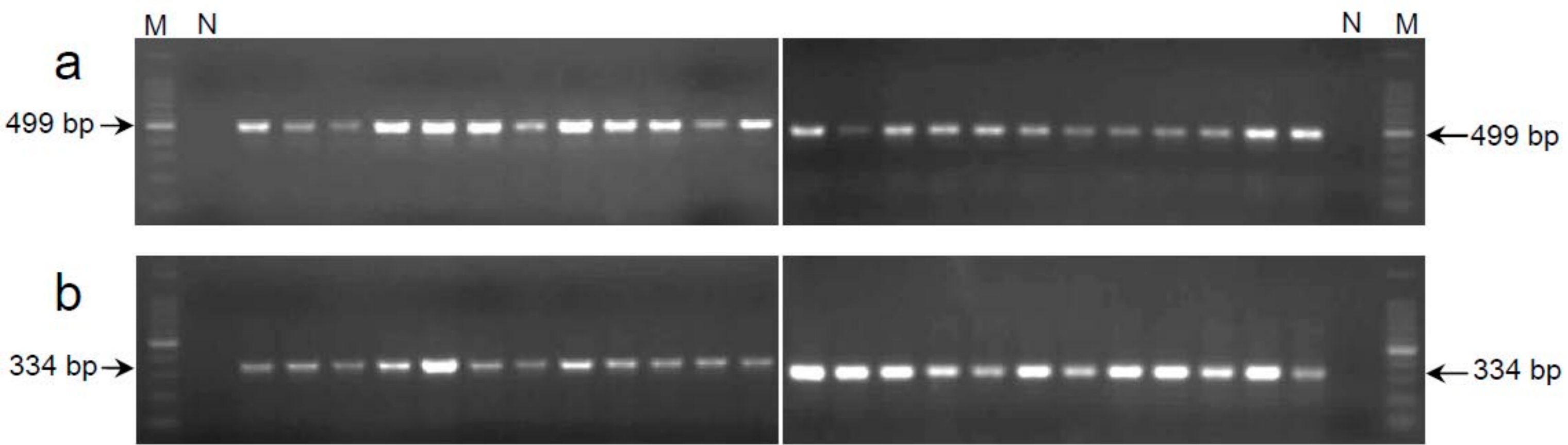

Supplement: Supplementary file 2 — Testification of TLR2 gene in C57BL/6 J mice by PCR. a PCR amplified products (499 bp) for TLR2 wild type. b PCR amplified products (334 bp) for TLR2 mutant. The data showed half samples of all. N, PCR no DNA template controls; M, 100 bp DNA ladder. (PDF 41 kb) [file 13071_2017_2318_MOESM2_ESM.pdf]
